# Spontaneous and Radiation-Induced Chromosome Aberrations in Primary Fibroblasts of Patients With Pediatric First and Second Neoplasms

**DOI:** 10.3389/fonc.2020.01338

**Published:** 2020-08-07

**Authors:** Sebastian Zahnreich, Alicia Poplawski, Carola Hartel, Lukas Stefan Eckhard, Danuta Galetzka, Thomas Hankeln, Markus Löbrich, Manuela Marron, Johanna Mirsch, Sylvia Ritter, Peter Scholz-Kreisel, Claudia Spix, Heinz Schmidberger

**Affiliations:** ^1^Department of Radiation Oncology and Radiation Therapy, University Medical Centre of the Johannes Gutenberg University Mainz, Mainz, Germany; ^2^Institute of Medical Biostatistics, Epidemiology and Informatics, University Medical Centre of the Johannes Gutenberg University Mainz, Mainz, Germany; ^3^Department of Biophysics, GSI Helmholtzzentrum für Schwerionenforschung GmbH, Darmstadt, Germany; ^4^Department of Orthopedic Surgery, University Medical Centre of the Johannes Gutenberg University Mainz, Mainz, Germany; ^5^Institute of Organismic and Molecular Evolution, Molecular Genetics and Genome Analysis, Johannes Gutenberg University Mainz, Mainz, Germany; ^6^Radiation Biology and DNA Repair, Technical University of Darmstadt, Darmstadt, Germany; ^7^Department of Epidemiological Methods and Etiologic Research, Leibniz Institute for Prevention Research and Epidemiology – BIPS, Bremen, Germany; ^8^German Childhood Cancer Registry, Institute of Medical Biostatistics, Epidemiology and Informatics, University Medical Centre of the Johannes Gutenberg University Mainz, Mainz, Germany

**Keywords:** childhood cancer, second primary malignancies, ionizing radiation, chromosome aberrations, radiation sensitivity, spontaneous chromosomal instability

## Abstract

The purpose of the present study was to investigate whether former childhood cancer patients who developed a subsequent secondary primary neoplasm (SPN) are characterized by elevated spontaneous chromosomal instability or cellular and chromosomal radiation sensitivity as surrogate markers of compromised DNA repair compared to childhood cancer patients with a first primary neoplasm (FPN) only or tumor-free controls. Primary skin fibroblasts were obtained in a nested case-control study including 23 patients with a pediatric FPN, 22 matched patients with a pediatric FPN and an SPN, and 22 matched tumor-free donors. Clonogenic cell survival and cytogenetic aberrations in Giemsa-stained first metaphases were assessed after X-irradiation in G1 or on prematurely condensed chromosomes of cells irradiated and analyzed in G2. Fluorescence *in situ* hybridization was applied to investigate spontaneous transmissible aberrations in selected donors. No significant difference in clonogenic survival or the average yield of spontaneous or radiation-induced aberrations was found between the study populations. However, two donors with an SPN showed striking spontaneous chromosomal instability occurring as high rates of numerical and structural aberrations or non-clonal and clonal translocations. No correlation was found between radiation sensitivity and a susceptibility to a pediatric FPN or a treatment-associated SPN. Together, the results of this unique case-control study show genomic stability and normal radiation sensitivity in normal somatic cells of donors with an early and high intrinsic or therapy-associated tumor risk. These findings provide valuable information for future studies on the etiology of sporadic childhood cancer and therapy-related SPN as well as for the establishment of predictive biomarkers based on altered DNA repair processes.

## Introduction

Over the past decades, the number of cancer survivors increased constantly due to earlier diagnosis as well as optimized and new oncologic therapies with increased effectiveness in local and systemic tumor control (1). However, the benefit of prolonged survival of cancer patients is compromised by an elevated risk for therapy-related adverse late-effects, with second primary neoplasms (SPN) representing the heaviest burden for the patients (2). This is of particular concern for the treatment of highly cancer-prone individuals with pediatric malignancies who are at the utmost risk for SPN because of high innate susceptibility, the clastogenic impact of anti-tumor treatments and prolonged survival after effectual cancer therapy with an average 5-year survival rate of about 80% (3). In childhood cancer patients, the successful control of a first primary neoplasm (FPN) by chemotherapy (CT) or radiation therapy (RT) increases the relative risk to develop a therapy-related SPN up to about 6-fold compared to the healthy population, corresponding to an incidence of more than 20% at 30 years after the diagnosis of the FPN (4–6). RT in particular, which is applied to treat more than 50% of all cancer patients during their clinical management, represents a high and established risk factor for therapy-related SPN (7). However, despite such correlations there is still a large variation in the individual susceptibility to treatment-induced SPN which has been attributed to genetic variation.

Pediatric cancers reflect a very heterogeneous group of disorders of mostly unidentified etiology. Only 5–10% of early-onset malignancies can be ascribed to known inherited or *de novo* familial mutations in high-penetrance predisposing genes (8). It is commonly assumed that genetic alterations in DNA repair and damage response pathways increase the inherent cancer risk and the vulnerability to adverse side-effects of oncologic therapies (9–11). However, the causalities for the vast majority of sporadic childhood cancers or an inherent susceptibility to iatrogenic SPN remain to be unraveled.

A clinical approach to identify individuals with an elevated cancer-proneness or hypersensitivity toward genotoxic anti-tumor therapies are functional bioassays which monitor the efficiency and accuracy of DNA repair in somatic cells after exposure to cytostatic drugs or ionizing radiation (IR). For this purpose, test systems have been developed to measure the efficiency and fidelity of DNA repair in lymphocytes or fibroblasts after *in vitro* IR exposure based on the quantification of chromosome aberrations or DNA double-strand break (DSB) repair foci [γH2AX or tumor protein 53 binding protein 1 (53BP1)] (12).

Common cytogenetic assays that are used to test for intrinsic chromosomal IR sensitivity investigate the rate of IR-induced chromosome aberrations in first metaphases after G0/1 exposure (G1 assay) or the frequency of chromatid aberrations in metaphases after irradiation of exponentially growing cells in G2 (G2 assay). The G2 assay allows for a prompter analysis of IR-induced cytogenetic damage since cells progress from G2 to mitosis within a few hours after irradiation. A variation of the G2 assay, which overcomes the problems of the IR-induced G2/M arrest and low mitotic indices, is drug-induced premature chromosome condensation in G2 (G2-PCC) (13). In most studies, a predisposition to RT-related severe normal tissue toxicities or cancer proneness correlated with a compromised repair of IR-induced DSBs and elevated rates of cytogenetic aberrations (14–31). Also, the level of spontaneous chromosome aberrations in normal somatic cells is considered a strong indicator of tumor incidence (32, 33). However, to identify patients who are at high risk for a pediatric FPN or a therapy-related SPN based on such surrogate biomarkers of compromised DNA repair and genome instability is still a major and unsolved clinical challenge.

To this end, we examined spontaneous chromosome aberrations as well as cellular and chromosomal IR sensitivity in primary skin fibroblasts obtained from a carefully matched case-control study nested in a cohort of childhood cancer survivors who were successfully treated for a sporadic FPN and either developed an SPN or not. Our findings will provide relevant clinical information whether sporadic and IR-induced chromosome aberrations and thus a limited DNA repair capacity in normal somatic cells can be used as a measure of risk assessment and stratification for the development of pediatric FPN or subsequent SPN.

## Materials and Methods

### Patients

FPN and SPN patients were registered at the German Childhood Cancer Registry at the University Medical Centre in Mainz, Germany (34) and were matched according to the entity of the FPN, the age at diagnosis of the FPN, the year of diagnosis of the FPN, the age at biopsy and sex. Participants with any entity of FPN or SPN were included. An overview of the patient characteristics is provided in Table 1. SPN donors had to survive for at least 1 year after the diagnosis of the SPN. Skin biopsies to obtain primary fibroblasts were collected from 23 patients suffering from a variety of pediatric FPN and no SPN, 22 patients with the same type of FPN, and an SPN after the successful treatment of the FPN and from 22 donors with no neoplasm (NN) after written informed consent. For each of the matched doublets of an FPN and an SPN case, a NN donor was matched according to sex and the age at biopsy within a 5-year age range. Biopsies of NN donors were sampled at the Department of Accident Surgery and Orthopedics at the University Medical Centre in Mainz, Germany during a planned surgery within the KiKme study (Marron et al., in review)^1^. Following this procedure, we obtained 18 matched triplets with a matched NN, FPN, and SPN donor each, two quadruplets with an additional FPN donor each, a quadruplet with an additional NN donor, and a single SPN donor (also indicated in Tables 2–**4** presenting the raw data of our analyses). The study was approved by the Ethics Committee of the Medical Association of Rhineland-Palatinate [No. 837.440.03 (4102) and No. 837.262.12(8363-F)].

**Table 1:** Overview of patient characteristics.

	**First primary neoplasms, *n* (%)**	**Second primary neoplasms, *n* (%)**
**Total**	45	22
**Female**	19 (42%)	10 (46%)
**Male**	26 (58%)	12 (54%)
**Age (years) at diagnosis**, ***median (range)***	5.0 (0–14)	15.5 (5–30)
<2	5 (11%)	
2–5	19 (42%)	
5–10	7 (16%)	2 (9%)
10–15	14 (31%)	7 (32%)
15–20		9 (41%)
20–25		2 (9%)
25–30		2 (9%)
**Calendar year at diagnosis**, ***median (range)***	1985 (1980–1998)	1997 (1985–2003)
1980–1985	19 (42%)	
1985–1990	13 (29%)	2 (9%)
1990–1995	7 (16%)	6 (27%)
1995–2000	6 (13%)	8 (36%)
2000–2005		6 (27%)
**Latency (years) between FPN and SPN**, ***median (range)***		8 (1–21)
1–5		2 (9%)
5–10		12 (55%)
10–15		2 (9%)
15–20		4 (18%)
20–25		2 (9%)
**Age (years) at skin biopsy**, ***median (range)***	25 (21–36)^a^	26 (20–40)
20–25	8 (35%)^a^	8 (36%)
25–30	13 (57%)^a^	12 (55%)
30–35	1 (4%)^a^	1 (5%)
35–40	1 (4%)^a^	1 (5%)
**Tumor entity according to ICC-3 code**		
**Leukemia (I)**		
Lymphoid leukemias (Ia)	21 (47%)	1 (5%)
Acute myeloid leukemias (Ib)	2 (4%)	
Myelodysplastic syndrome and other myeloproliferative diseases (Id)	1 (2%)	1 (5%)
**Lymphoma (II)**		
Hodgkin lymphomas (IIa)	8 (18%)	1 (5%)
Non-Hodgkin lymphomas (except Burkitt lymphoma) (IIb)		5 (23%)
Burkitt lymphoma (IIc)	2 (4%)	
**CNS and miscellaneous intracranial and intraspinal neoplasms (III)**		
Ependymomas and choroid plexus tumor (IIIa)		1 (5%)
Astrocytomas (IIIb)		1 (5%)
Medulloblastoma (IIIc, 1.)	2 (4%)	
Other specified intracranial and intraspinal neoplasms (IIIe, 5.)		2 (9%)
**Neuroblastoma and other peripheral nervous cell tumors (IV)**		
Neuroblastoma and ganglioneuroblastoma (IVa)	2 (4%)	
**Retinoblastoma (V)**	2 (4%)	
**Renal tumors (VI)**		
Nephroblastoma and other non-epithelial renal tumors (VIa)	2 (4%)	
**Soft tissue and other extraosseous sarcomas (IX)**		
Rhabdomyosarcomas (IXa)	3 (7%)	
Other specified soft tissue sarcomas (IXd)		1 (5%)
**Other malignant epithelial neoplasms and malignant melanomas (XI)**		
Thyroid carcinomas (XIb)		6 (27%)
Carcinomas of salivary glands (XIf, 1.)		3 (14%)
**Oncologic therapies**		
CT– RT–	1 (2%)	8 (36%)
CT+ RT–	12 (27%)	5 (23%)
CT– RT+	1 (2%)	5 (23%)
CT+ RT+	31 (69%)	4 (18%)

*First primary neoplasms comprise all cancer patients if not stated otherwise. Tumor entities were classified according to (35)*.

a *Cancer patients with a first primary neoplasm and no subsequent second primary neoplasm only*.

*CT, chemotherapy; RT, radiation therapy including radioiodine-therapy for papillary thyroid cancer*.

**Table 2:** Clonogenic survival after X-ray exposure of fibroblasts in G1.

**Donor**	**Surviving fraction**		
	**2 Gy**	**4 Gy**	**6 Gy**
NN1	0.621 ± 0.070	0.188 ± 0.032	0.065 ± 0.019
FPN1	0.583 ± 0.144	0.160 ± 0.032	0.044 ± 0.025
SPN1	0.589 ± 0.092	0.220 ± 0.028	0.046 ± 0.010
NN2	0.333 ± 0.110	0.201 ± 0.079	-
FPN2	0.490 ± 0.031	0.260 ± 0.031	0.051 ± 0.022
SPN2	0.473 ± 0.179	0.188 ± 0.060	0.033 ± 0.003
NN3	0.456 ± 0.046	0.358 ± 0.102	0.070 ± 0.038
FPN3	0.917 ± 0.011	0.446 ± 0.018	0.095 ± 0.008
SPN3	0.483 ± 0.059	0.312 ± 0.024	0.119 ± 0.001
NN4	0.581 ± 0.031	0.266 ± 0.016	0.080 ± 0.014
FPN4	0.559 ± 0.049	0.202 ± 0.036	0.072 ± 0.010
SPN4	0.512 ± 0.026	0.222 ± 0.021	0.059 ± 0.012
SPN5	0.375 ± 0.225	0.363 ± 0.115	0.090 ± 0.079
NN6	0.541 ± 0.016	0.247 ± 0.013	0.116 ± 0.006
FPN6	0.365 ± 0.113	0.223 ± 0.027	0.119 ± 0.031
SPN6	0.588 ± 0.017	0.307 ± 0.010	0.134 ± 0.010
NN7	0.489 ± 0.059	0.292 ± 0.009	0.137 ± 0.007
FPN7	0.523 ± 0.072	0.222 ± 0.024	0.100 ± 0.005
SPN7	0.493 ± 0.036	0.186 ± 0.020	0.092 ± 0.007
NN8	0.500 ± 0.164	0.329 ± 0.035	0.116 ± 0.018
FPN8	0.452 ± 0.128	0.149 ± 0.036	0.055 ± 0.027
FPN8a	0.448 ± 0.047	0.168 ± 0.022	0.038 ± 0.004
SPN8	0.722 ± 0.106	0.278 ± 0.042	0.010 ± 0.006
NN9	0.677 ± 0.162	0.399 ± 0.048	0.193 ± 0.034
FPN9	0.393 ± 0.110	0.157 ± 0.016	-
SPN9	0.344 ± 0.116	0.172 ± 0.033	0.059 ± 0.020
NN10	0.478 ± 0.049	0.168 ± 0.061	0.112 ± 0.019
FPN10	0.524 ± 0.078	0.263 ± 0.042	0.097 ± 0.010
SPN10	0.425 ± 0.064	0.331 ± 0.037	0.128 ± 0.030
NN11	0.357 ± 0.076	0.140 ± 0.017	0.056 ± 0.017
FPN11	0.604 ± 0.088	0.212 ± 0.066	0.115 ± 0.008
SPN11	0.548 ± 0.129	0.274 ± 0.035	0.097 ± 0.014
NN12	0.374 ± 0.011	0.179 ± 0.020	0.041 ± 0.009
FPN12	0.779 ± 0.104	0.209 ± 0.036	0.073 ± 0.054
FPN12a	0.385 ± 0.037	0.134 ± 0.006	0.044 ± 0.011
SPN12	0.407 ± 0.028	0.197 ± 0.024	0.041 ± 0.009
NN13	0.684 ± 0.052	0.234 ± 0.028	0.074 ± 0.004
FPN13	0.653 ± 0.128	0.215 ± 0.019	0.086 ± 0.010
SPN13	0.239 ± 0.042	0.120 ± 0.008	0.023 ± 0.040
NN14	0.613 ± 0.032	0.265 ± 0.033	0.090 ± 0.005
FPN14	0.489 ± 0.032	0.212 ± 0.029	0.106 ± 0.026
SPN14	0.407 ± 0.070	0.222 ± 0.031	0.061 ± 0.024
NN15	0.178 ± 0.031	0.081 ± 0.013	0.012 ± 0.011
FPN15	0.920 ± 0.466	0.349 ± 0.025	0.076 ± 0.036
SPN15	0.568 ± 0.070	0.197 ± 0.056	0.081 ± 0.043
NN16	0.887 ± 0.054	0.365 ± 0.042	0.136 ± 0.027
FPN16	0.386 ± 0.164	0.175 ± 0.092	0.022 ± 0.007
SPN16	0.587 ± 0.035	0.266 ± 0.035	0.064 ± 0.010
NN17	0.500 ± 0.107	0.362 ± 0.101	0.109 ± 0.025
FPN17	0.507 ± 0.013	0.212 ± 0.023	0.067 ± 0.009
SPN17	0.682 ± 0.074	0.324 ± 0.059	0.106 ± 0.013
NN18	0.658 ± 0.111	0.097 ± 0.023	0.017 ± 0.013
FPN18	0.647 ± 0.280	0.390 ± 0.046	0.018 ± 0.018
SPN18	0.631 ± 0.043	0.284 ± 0.022	0.093 ± 0.008
NN19	0.551 ± 0.033	0.299 ± 0.026	0.135 ± 0.012
FPN19	0.628 ± 0.064	0.286 ± 0.040	0.036 ± 0.006
SPN19	0.737 ± 0.178	0.204 ± 0.057	0.044 ± 0.020
NN20	0.804 ± 0.197	0.331 ± 0.076	0.127 ± 0.016
FPN20	0.743 ± 0.086	0.323 ± 0.058	0.136 ± 0.016
SPN20	0.950 ± 0.108	0.339 ± 0.015	0.074 ± 0.007
NN21	0.521 ± 0.007	0.293 ± 0.011	0.063 ± 0.010
FPN21	0.628 ± 0.096	0.236 ± 0.090	0.096 ± 0.036
SPN21	0.670 ± 0.075	0.292 ± 0.009	0.130 ± 0.025
NN22	0.417 ± 0.036	0.234 ± 0.095	0.055 ± 0.023
NN22a	n.a.	n.a.	n.a.
FPN22	0.683 ± 0.136	0.233 ± 0.026	0.059 ± 0.013
SPN22	0.551 ± 0.092	0.203 ± 0.011	0.056 ± 0.015

*Data are presented as the mean fraction of surviving cells and the standard deviation of three technical replicates from one experiment for each donor*.

*NN, no neoplasm; FPN, first primary neoplasm; SPN, second primary neoplasm*.

### Cell Culture and Irradiation

To obtain primary human skin fibroblasts from cancer patients, biopsies were taken on the inside of the cubital region and for NN donors at the site of the planned surgery. Biopsies were dissected and kept in rich cell culture medium (Amniogrow, CytoGen GmbH, Wetzlar, Germany) in a humidified incubator at 37°C and 5% CO_2_ to allow for outgrowth and expansion of primary fibroblasts. After the first passage, cells were cultured in Dulbecco‘s Minimal Essential Medium (Sigma-Aldrich, St. Louis, USA) containing 1% non-essential amino acids (Biochrom, Berlin, Germany), 15% fetal bovine serum (Biochrom, Berlin, Germany), and 1% penicillin/streptomycin (Biochrom, Berlin, Germany). Passaging was done using 0.05% trypsin with 0.1% ethylene-diamine-tetra-acetate (Biochrom, Berlin, Germany). After further expansion, cells were cryopreserved in liquid nitrogen. Only cells with <20 population doublings were used for experiments.

To irradiate fibroblasts selectively in G1 for clonogenic survival and the cytogenetic G1 assay, cells were synchronized by contact inhibition. To obtain confluent cultures, fibroblasts were seeded at a density of 9,000 cells/cm^2^ in cell culture dishes with a diameter of 10 cm (Greiner Bio-One GmbH, Frickenhausen, Germany) and were allowed to grow at least for 14 days with a medium change every 4 days. Synchronization in G1 was confirmed by flow cytometric cell cycle analysis revealing that more than 90% of the population was in G1 when the cells were exposed to X-rays. To irradiate and analyze cells selectively in G2 (G2 assay), 9,000 cells/cm^2^ were seeded in cell culture dishes with a diameter of 10 cm (Greiner Bio-One GmbH, Frickenhausen, Germany). Two days later exponentially growing cells were exposed to X-rays.

Irradiation was performed with a D3150 X-Ray Therapy System (Gulmay Ltd., Surrey, UK) at 140 kV and a dose rate of 3.6 Gy/min at room temperature. Control cells were sham-irradiated, i.e., kept at the same conditions in the radiation device control room.

### Clonogenic Survival

To determine the clonogenic survival of G1 fibroblasts, confluent cultures were irradiated. Cells were harvested 24 h after irradiation and seeded in culture dishes with a diameter of 10 cm (Greiner Bio-One GmbH, Frickenhausen, Germany). 13, 26, 52, and 65 cells/cm^2^ were plated after exposure to 0, 2, 4, and 6 Gy X-rays, respectively, with three technical replicates per IR dose. After incubation for 14 days to allow for the formation of colonies, cells were fixed, stained, and scored according to the protocol of Puck and Marcus (36), and survival curves were established considering the plating efficiencies of sham-irradiated controls. The average numbers of surviving cells per technical replicate counted as colonies with at least 50 cells after exposure to 0, 2, 4, and 6 Gy X-rays from all available samples were 59.3 ± 39.3, 65.6 ± 39.9, 58.0 ± 39.9, and 25.6 ± 20.7 (mean ± standard deviation), respectively.

### Cytogenetic Assays

#### G1 Assay

Confluent fibroblasts were harvested 24 h after exposure to 3 Gy X-rays and seeded at a density of 9,000 cells/cm^2^ in 75 cm^2^ cell culture flasks (Greiner Bio-One GmbH, Frickenhausen, Germany). After 24 h, 0.02 μg/ml Colcemid (Roche, Basel, Switzerland) was added to collect only first metaphases after IR exposure as confirmed by BrdU labeling and fluorescence plus Giemsa staining performed according to Perry and Wolff (37). This approach was further validated by measuring the cell cycle progression after delayed plating by flow cytometry (Supplementary Figure S1). Forty-eight hours after delayed plating cells were trypsinized, chromosome spreads were prepared, and Giemsa-stained as described previously (38). This technique allows the detection of unstable chromosome aberrations which are categorized as being not transmissible to daughter cells comprising dicentric chromosomes, centric rings, and excess acentric fragments as well as chromatid breaks and exchanges (radials). For each sample 100 complete diploid metaphases were analyzed according to the criteria defined by Savage (39). The fraction of tetraploid cells was calculated as the ratio of metaphases with 92 chromosomes divided by the total number of all analyzed diploid and tetraploid metaphases. To assess the proportion of tetraploid cells on average 506 metaphases were scored for each sample.

#### G2 Assay

For the analysis of chromatid aberrations in cells exposed to 1 Gy X-rays in G2, 2.25 h after irradiation 50 nM calyculin A (LC Laboratories, Woburn, US) was added for 45 min to the cultures to induce G2-PCC. Detached cells were collected and chromosome preparation and Giemsa staining were performed as described previously (38). Chromatid aberrations were scored as breaks, gaps, and exchanges (radials) in G2-PCCs with at least 46 chromosome pieces. Gaps, which are usually considered to be achromatic lesions rather than true chromatid discontinuities, were included due to the obscure structure of chromosomes after G2-PCC. Chromatid exchanges were rare and scored as one aberration. Isochromatid breaks were scored as 2 chromatid breaks. Generally, 100 G2-PCCs were analyzed for each control sample and 50 G2-PCCs were analyzed for each irradiated sample.

### Cytogenetic Data

The yield of spontaneous aberrations in sham-irradiated cells was subtracted from that in irradiated samples. If the desired number of metaphases or G2-PCCs was not achieved due to poor proliferation even in repeated experiments, all available metaphases or G2-PCCs were used for the analysis. The exact numbers of analyzed cells are provided in Tables 3, 4.

**Table 3:** Spontaneous and radiation-induced chromosome aberrations per cell in first post-exposure metaphases collected 48 h after irradiation of fibroblasts in G1 with 3 Gy X-rays (G1 assay).

**Donor**	**0 Gy**									**3 Gy**							
	**Cells**	**Aberrant cells (%)**	**Aberrations**	**dic**	***r***	**ace**	**ctb**	**cte**	**4N (%)**	**Cells**	**Aberrant cells (%)**	**Aberrations**	**dic**	***r***	**ace**	**ctb**	**cte**
NN1	100	17.0	0.26	0.01	–	0.10	0.15	–	3.6	100	61.0	0.77	0.36	0.03	0.35	0.01	0.02
FPN1	100	7.0	0.08	0.02	–	0.05	0.01	–	1.5	100	54.0	0.59	0.36	0.02	0.21	–	–
SPN1	100	15.0	0.21	0.07	–	0.10	0.03	0.01	0.8	71	60.6	0.59	0.18	0.03	0.37	0.01	–
NN2	100	4.0	0.04	–	0.02	–	0.02	–	0.6	100	49.0	0.70	0.39	–	0.28	0.03	–
FPN2	100	8.0	0.10	–	–	0.06	0.04	–	1.2	100	52.0	0.66	0.36	0.02	0.28	–	–
SPN2	100	4.0	0.05	0.01	–	0.03	0.01	–	1.2	100	48.0	0.66	0.34	0.02	0.29	–	0.01
NN3	100	9.0	0.12	–	–	0.04	0.08	–	1.2	100	47.0	0.54	0.31	0.01	0.22	–	–
FPN3	100	1.0	0.01	–	–	0.01	–	–	0.4	100	44.0	0.60	0.32	0.01	0.27	–	–
SPN3	100	7.0	0.09	–	–	0.07	0.02	–	2.0	100	49.0	0.55	0.35	0.02	0.17	0.01	–
NN4	100	1.0	0.01	–	–	–	0.01	–	2.3	57	63.2	0.74	0.23	0.04	0.47	–	–
FPN4	100	3.0	0.03	–	–	0.01	0.02	–	2.1	100	56.0	0.87	0.40	–	0.44	0.03	–
SPN4	100	10.0	0.10	0.01	0.01	0.04	0.04	–	1.0	100	57.0	0.73	0.38	–	0.35	–	–
SPN5	100	2.0	0.02	–	–	0.01	0.01	–	1.3	100	57.0	0.66	0.38	0.01	0.27	–	–
NN6	100	3.0	0.04	–	–	0.03	0.01	–	0.2	100	58.0	0.76	0.26	0.04	0.42	0.04	–
FPN6	n.a				–					n.a.							
SPN6	100	13.0	0.18	–	–	0.16	0.02	–	0.7	100	50.0	0.58	0.27	0.01	0.30	–	–
NN7	100	2.0	0.02	–	–	0.01	0.01	–	1.0	100	65.0	0.79	0.38	0.03	0.38	–	–
FPN7	100	2.0	0.02	–	–	0.02	–	–	1.5	100	49.0	0.65	0.35	0.02	0.26	0.02	–
SPN7	58	12.1	0.14	0.02	0.02	0.07	0.03	–	1.6	76	60.5	0.80	0.35	–	0.44	0.01	–
NN8	100	6.0	0.06	–	–	0.06	–	–	0.8	100	40.0	0.47	0.26	–	0.21	–	–
FPN8	100	7.0	0.07	0.04	–	0.03	–	–	1.8	100	57.0	0.77	0.40	–	0.31	0.06	–
FPN8a	100	–	–	–	–	–	–	–	1.5	100	61.0	0.94	0.34	0.01	0.57	0.01	–
SPN8	100	1.0	0.01	–	–	0.01	–	–	2.3	100	52.0	0.64	0.35	0.02	0.27	–	–
NN9	100	14.0	0.16	0.01	–	0.07	0.08	–	0.4	100	46.0	0.48	0.25	0.02	0.21	–	–
FPN9	100	17.0	0.18	0.04	–	0.14	–	–	1.7	100	56.0	0.48	0.29	0.02	0.17	–	–
SPN9	100	2.0	0.02	–	–	0.02	–	–	4.0	47	48.9	0.58	0.28	0.02	0.28	–	–
NN10	100	3.0	0.03	–	–	0.01	0.02	–	2.8	100	50.0	0.64	0.30	0.01	0.31	0.02	–
FPN10	100	9.0	0.09	–	–	0.06	0.03	–	2.0	n.a							
SPN10	100	1.0	0.02	–	–	0.02	–	–	1.1	n.a							
NN11	100	18.0	0.23	0.03	–	0.07	0.13	–	2.2	100	56.0	0.76	0.39	0.03	0.34	–	–
FPN11	78	7.7	0.09	–	–	0.09	–	–	–	100	56.0	0.61	0.26	0.06	0.28	0.01	–
SPN11	100	6.0	0.07	–	–	0.05	0.02	–	1.0	100	57.0	0.68	0.46	0.01	0.21	–	–
NN12	100	2.0	0.02	–	–	0.02	–	–	1.3	88	51.1	0.61	0.28	0.02	0.26	0.03	–
FPN12	100	3.0	0.03	0.01	–	0.02	–	–	0.2	100	59.0	0.80	0.36	–	0.43	0.01	–
FPN12a	100	12.0	0.14	–	–	0.03	0.09	0.02	2.5	29	65.5	0.90	0.45	–	0.45	–	–
SPN12	100	18.0	0.25	–	–	0.13	0.12	–	1.8	100	54.0	0.56	0.35	0.03	0.18	–	–
NN13	100	8.0	0.09	0.01	–	0.06	0.02	–	1.8	100	48.0	0.59	0.28	0.01	0.30	–	–
FPN13	100	9.0	0.01	–	–	0.07	0.03	–	1.7	100	49.0	0.53	0.29	–	0.24	–	–
SPN13	88	19.3	0.22	0.02	–	0.07	0.13	–	3.3	100	70.0	0.87	0.30	0.01	0.56	–	–
NN14	100	5.0	0.06	0.02	–	0.03	0.01	–	0.3	100	59.0	0.74	0.35	0.01	0.33	0.05	–
FPN14	100	15.0	0.17	0.08	–	0.05	0.04	–	0.2	94	62.8	0.71	0.41	–	0.30	–	–
SPN14	100	5.0	0.06	0.02	–	0.03	0.01	–	1.0	100	56.0	0.72	0.34	0.01	0.32	0.05	–
NN15	100	6.0	0.06	0.01	–	0.02	0.03	–	0.2	n.a							
FPN15	74	10.8	0.12	0.01	–	0.10	0.01	–	0.2	n.a							
SPN15	100	17.0	0.21	0.03	–	0.14	0.04	–	0.2	66	29.0	0.41	0.24	0.02	0.14	–	–
NN16	100	6.0	0.06	0.02	–	0.04	–	–	1.4	100	55.0	0.80	0.45	–	0.35	–	–
FPN16	100	13.0	0.15	0.05	–	0.09	0.01	–	7.4	100	58.0	0.79	0.45	0.03	0.27	0.04	–
SPN16	100	3.0	0.03	–	–	0.03	–	–	1.6	96	51.0	0.77	0.35	–	0.42	–	–
NN17	100	6.0	0.06	–	–	0.06	–	–	2.0	100	50.0	0.58	0.33	–	0.25	–	–
FPN17	100	16.0	0.22	–	–	0.17	0.05	–	1.2	100	47.0	0.45	0.25	–	0.20	–	–
SPN17	100	10.0	0.11	0.02	–	0.08	0.01	–	1.0	59	50.9	0.64	0.29	0.03	0.29	0.02	–
NN18	100	9.0	0.09	–	–	0.06	0.03	–	1.2	100	38.0	0.49	0.29	–	0.20	–	–
FPN18	100	1.0	0.01	–	–	0.01	–	–	8.8	100	48.0	0.63	0.31	0.02	0.30	–	–
SPN18	100	5.0	0.09	0.01	–	0.08	–	–	1.2	45	46.7	0.53	0.28	0.07	0.16	0.02	–
NN19	100	3.0	0.03	0.01	–	0.01	0.01	–	0.8	100	51.0	0.70	0.40	0.01	0.29	–	–
FPN19	100	7.0	0.07	–	–	0.07	–	–	0.6	100	59.0	0.87	0.45	0.01	0.41	–	–
SPN19	100	1.0	0.01	0.01	–	–	–	–	0.5	100	52.0	0.69	0.39	–	0.30	–	–
NN20	100	11.0	0.11	–	–	0.08	0.03	–	0.6	100	40.0	0.43	0.23	0.01	0.19	–	–
FPN20	100	12.0	0.12	–	–	0.09	0.03	–	0.8	100	45.0	0.52	0.31	0.03	0.18	–	–
SPN20	100	3.0	0.05	0.01	–	0.03	0.01	–	1.8	87	50.6	0.72	0.40	–	0.32	–	–
NN21	100	9.0	0.10	0.04	–	0.05	0.10	–	5.4	100	54.0	0.65	0.36	–	0.29	–	–
FPN21	100	7.0	0.07	0.03	–	0.04	–	–	4.0	100	57.0	0.61	0.26	0.01	0.34	–	–
SPN21	100	21.0	0.30	0.01	–	0.11	0.16	0.02	5.1	100	44.0	0.43	0.29	0.01	0.13	–	–
NN22	100	16.0	0.18	0.04	–	0.06	0.07	0.01	2.0	100	53.0	0.63	0.35	0.03	0.25	–	–
NN22a	100	5.0	0.07	–	–	0.07	–	–	0.8	100	54.0	0.63	0.28	0.03	0.31	0.01	–
FPN22	100	5.0	0.06	0.01	–	0.05	–	–	1.2	n.a.							
SPN22	100	3.0	0.03	–	–	0.03	–	–	2.2	100	37.0	0.40	0.24	0.04	0.12	–	–

*For some donors the cytogenetic analysis was not available (n.a.) due to an insufficient mitotic index*.

*NN, no neoplasm; FPN, first primary neoplasm; SPN, second primary neoplasm; dic, dicentric chromosome; r, centric ring chromosome; ace, excess acentric fragment; ctb, chromatid break; cte, chromatid exchange (radial); 4N, fraction of tetraploid mitoses*.

**Table 4:** Spontaneous and radiation-induced chromatid aberrations per G2-PCC 3 h after irradiation of exponentially growing fibroblasts with 1 Gy X-rays (G2 assay).

**Donor**	**0 Gy**					**1 Gy**				
	**Cells**	**Aberrant cells (%)**	**Aberrations**	**ctb/gaps**	**cte**	**Cells**	**Aberrant cells (%)**	**Aberrations**	**ctb/gaps**	**cte**
NN1	100	45.0	1.09	1.09	–	50	96.0	6.03	6.03	–
FPN1	100	44.0	0.98	0.98	–	50	100	5.74	5.74	–
SPN1	100	44.0	1.10	1.10	–	50	100	5.64	5.64	–
NN2	100	20.0	0.33	0.33	–	50	100	6.85	6.79	0.06
FPN2	96	20.8	0.62	0.62	–	49	100	6.92	6.92	–
SPN2	100	28.0	0.46	0.46	–	54	100	6.50	6.50	–
NN3	100	27.0	0.50	0.50	–	50	92.0	5.96	5.92	0.04
FPN3	100	31.0	0.59	0.59	–	50	96.0	4.25	4.25	–
SPN3	100	40.0	0.65	0.65	–	50	100	6.05	6.05	–
NN4	100	30.0	0.65	0.64	0.01	50	98.0	6.70	6.64	0.03
FPN4	100	50.0	1.26	1.26	–	50	100	4.32	4.28	0.02
SPN4	100	38.0	0.56	–	–	50	100	6.50	6.34	0.08
SPN5	100	14.0	0.17	0.17	–	38	100	5.47	5.44	0.03
NN6	100	44.0	0.98	0.98	–	50	100	6.18	6.14	0.04
FPN6	n.a.					n.a.				
SPN6	100	48.0	1.60	1.58	0.02	50	96.0	5.92	5.92	–
NN7	100	22.0	0.40	0.40	–	50	98.0	5.36	5.36	–
FPN7	83	33.7	0.69	0.69	–	46	100	5.16	5.16	–
SPN7	82	29.3	0.62	0.62	–	33	100	5.83	5.77	0.06
NN8	100	31.0	0.46	0.44	0.02	50	100	5.28	5.28	-
FPN8	100	31.0	0.61	0.58	0.03	50	100	7.83	7.68	0.15
FPN8a	100	26.0	0.33	0.32	0.01	50	100	6.50	6.50	-
SPN8	100	34.0	0.65	0.65	–	50	100	6.93	6.81	0.06
NN9	100	32.0	0.58	0.58	–	n.a.				
FPN9	100	18.0	0.43	0.43	–	23	100	5.57	5.57	–
SPN9	100	10.0	0.13	0.13	–	50	100	5.23	5.19	0.04
NN10	100	21.0	0.42	0.42	–	50	98.0	5.28	5.24	0.04
FPN10	100	40.0	0.78	0.78	–	50	100	5.70	5.68	0.02
SPN10	52	9.0	0.35	0.35	–	n.a.				
NN11	84	28.6	0.73	0.73	–	50	100	6.75	6.69	0.06
FPN11	74	48.7	0.96	0.96	–	49	98.0	5.60	5.60	–
SPN11	100	39.0	0.94	0.94	–	50	100	5.02	5.02	–
NN12	100	16.0	0.31	0.31	–	50	100	4.83	4.83	–
FPN12	100	36.0	0.62	0.62	–	50	100	5.18	5.18	–
FPN12a	100	47.0	0.85	0.78	0.07	50	100	5.69	5.64	0.05
SPN12	100	46.0	1.20	1.16	0.03	38	100	5.55	5.55	-
NN13	100	51.0	1.06	1.04	0.02	50	98.0	4.78	4.74	0.04
FPN13	100	41.0	1.05	1.02	0.03	50	100	6.06	6.06	–
SPN13	100	20.0	0.30	0.30	–	50	100	5.84	5.72	0.12
NN14	100	38.0	0.54	0.53	0.01	50	100	6.44	6.37	0.07
FPN14	100	31.0	0.77	0.74	0.03	50	100	5.73	5.68	0.05
SPN14	100	30.0	0.68	0.68	–	32	100	5.45	5.38	0.06
NN15	100	31.0	0.74	0.69	0.05	50	100	6.11	6.11	–
FPN15	100	8.0	0.11	–	–	50	100	5.35	5.35	–
SPN15	98	24.5	0.39	0.39	–	48	100	5.57	5.47	0.10
NN16	100	46.0	1.22	1.22	–	49	98.0	7.64	7.62	0.02
FPN16	n.a.				–	n.a.				
SPN16	100	53.0	1.18	1.18	–	50	98.0	5.32	5.32	–
NN17	100	19.0	0.49	0.48	0.01	50	100	5.67	5.60	0.07
FPN17	100	42.0	1.37	1.31	0.06	50	100	8.59	8.47	0.12
SPN17	100	52.0	1.23	1.14	0.09	50	100	9.39	9.22	0.17
NN18	87	13.8	0.17	0.17	–	50	98.0	6.15	6.05	0.10
FPN18	100	40.0	1.13	1.01	0.03	50	100	6.32	6.25	0.07
SPN18	100	41.0	0.65	0.65	–	50	100	5.37	5.35	0.02
NN19	100	27.0	0.43	0.43	–	50	100	5.47	5.43	0.04
FPN19	100	27.0	0.39	0.39	–	50	100	4.83	4.77	0.06
SPN19	67	25.4	0.37	0.37	–	49	100	4.99	4.95	0.04
NN20	100	18.0	0.39	0.39	–	50	100	4.51	4.49	0.02
FPN20	100	36.0	1.04	0.96	0.08	50	100	6.58	6.48	0.10
SPN20	100	24.0	0.55	0.53	0.02	50	98.0	6.03	5.95	0.08
NN21	100	54.0	2.16	2.02	0.07	50	100	4.89	4.86	0.03
FPN21	100	41.0	0.82	0.79	0.03	37	100	6.48	6.32	0.16
SPN21	100	28.0	0.66	0.64	0.02	50	100	4.90	4.82	0.08
NN22	100	36.0	0.76	0.75	0.01	50	100	5.93	5.93	–
NN22a	100	22.0	0.48	0.48	–	50	98.0	5.47	5.47	–
FPN22	100	42.0	0.91	0.87	0.04	50	100	5.89	5.85	0.04
SPN22	73	34.3	0.55	0.55	–	56	100	6.29	6.29	–

*For some donors the cytogenetic analysis was not available (n.a.) due to poor proliferation and an insufficient G2-index*.

*NN, no neoplasm; FPN, first primary neoplasm; SPN, second primary neoplasm; ctb, chromatid break; cte, chromatid exchange (radial)*.

### Three- and Twenty-Four-Color Fluorescence *in situ* Hybridization

To detect transmissible aberrations in metaphase spreads obtained in the G1 assay, e.g., translocations or insertions which are not detected by the monochromic Giemsa method, fluorescence *in situ* hybridization (FISH) was performed for selected donors. Three-color FISH with a commercial cocktail of whole chromosome probes 1, 2, and 4 was conducted following the manufacturer's protocol (Metasystems, Altlussheim, Germany). Images of metaphases were obtained using an Axioimager1 microscope and the AxioVision software (Carl Zeiss AG, Oberkochen, Germany). Structural aberrations were scored only when painted chromosomes were involved. Analysis by 24-multicolor FISH (mFISH) which is capable of providing more detailed cytogenetic information (40) was performed as described previously (41). Slides were hybridized with a 24XCyte mFISH kit according to the manufacturer's protocol (Metasystems, Altlussheim, Germany). Captured images of metaphases were obtained with a Zeiss Axio-Imager Z2 microscope (Zeiss, Jena, Germany) and processed and analyzed with the Isis software (In Situ Imaging Systems, Metasystems, Germany). Structural aberrations were classified according to the mPAINT system (42). In brief, basic aberration forms such as translocations (t), insertions (ins), or dicentric chromosomes (dic) are described. Chromosome fragments containing a centromere are indicated by an apostrophe. An uppercase “T” indicates a truncated centric element which has become visibly shortened. For example, a reciprocal translocation between chromosomes 1 and 2 is designated “t (1′-2) (2′-1).” Identical chromosome aberrations were termed clonal if they were present in at least two metaphases of one sample. For each sample 100 diploid metaphases were analyzed.

### Data and Statistical Analysis

The clonogenic survival after IR exposure was analyzed using a linear mixed model with the fixed variables dose, dose^2^, group (NN, FPN, or SPN) and intercept including the patient as a random effect. The model was fitted using the lmer function in lme4 R package (43). Aberrations scored in the G1 and G2 assay were analyzed separately using the R package glmmTMB (44). Mixed models were fitted to estimate the effect of dose, group (NN, FPN, or SPN), a previous RT or CT, gender, and tumor entity on the number of aberrations. The negative binomial model with the patient as a random variable fitted best. Adding the matching group as an additional random variable did not improve the model. Age, sex, and tumor entity showed no significant impact on aberrations and were therefore excluded from the final model. The relationship between two variables was analyzed using Pearson's test and is provided as the correlation coefficient (*r*). All levels of significance were set at p < 0.05. Average rates of chromosome aberrations or the fraction of surviving cells of pooled donors of the different study populations are provided as the mean ± standard deviation.

## Results

### Patient Characteristics

An overview of the summarized characteristics of cancer patients is provided in Table 1. Total numbers of 23 cases with FPN only and 22 cases with the same FPN and a subsequent SPN as well as 22 NN donors were enclosed in the study. Hematopoietic and lymphoid cancers represented the majority of FPN (76%) and only a minor fraction of SPN (36%). Information on oncologic therapies is provided to the best of our knowledge based on the documentation of treating physicians voluntarily and on patient-based self-reports during a medical interview. All FPNs except for two retinoblastomas were treated by CT. However, since no matching of oncologic therapies has been performed between the corresponding FPN and SPN cases, differences in the application of RT were noted as follows: Four SPN and two FPN cases received radiochemotherapy compared to their respective FPN and SPN counterparts treated with CT only. In at least two other cases RT was administered to different or unknown anatomic regions. One FPN donor was treated by surgery only for unilateral retinoblastoma whereas the matched SPN case received RT only for bilateral retinoblastoma. One FPN and six SPN patients received a bone marrow or stem cell transplant during therapy without preconditioning by total body RT. For all patients, RT was administered locally to the site of a solid tumor, as cranial or craniospinal irradiation for leukemia, mainly at the thoracic and neck region for lymphoma or as radioiodine-therapy for papillary thyroid cancer. According to the common RT plans for the enclosed tumor entities, the treatment fields of partial-body RT did not involve the site of skin biopsy near the cubital region. Due to the retrospective nature of the study with an inevitable long follow-up, skin biopsies were collected from young adults on average 20 years after the diagnosis of the pediatric FPN and on average 10 years after the diagnosis of the adolescent SPN.

### Clonogenic Survival

**Figure 1:**
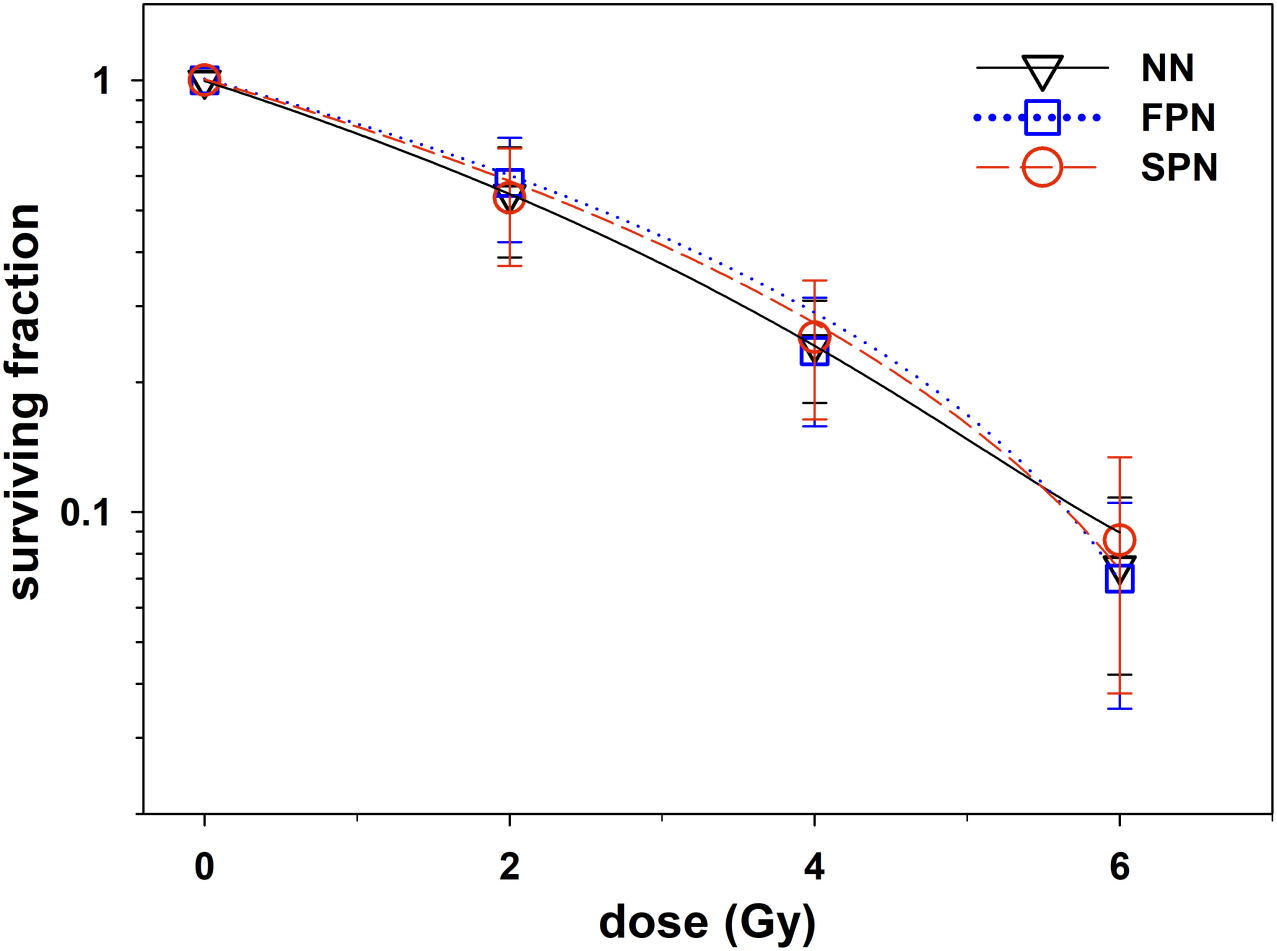
Clonogenic survival of primary skin fibroblasts from donors with no neoplasm (NN, *n* = 22), only a first primary neoplasm (FPN, *n* = 23) or an FPN and a subsequent second primary neoplasm (SPN, *n* = 21) after exposure to X-rays. Error bars represent the standard deviation. All lines were fitted by a linear-quadratic function.

The cellular IR sensitivity of primary fibroblasts was measured as clonogenic survival after X-ray exposure of cells in G1. The summarized results for FPN, SPN, and NN donors are presented in Figure 1 and raw data for each donor is provided in Table 2. The average plating efficiencies of NN, FPN, and SPN donors were similar with fractions of 6.4 ± 3.9%, 4.7 ± 3.4%, and 6.8 ± 3.9% of cells forming colonies, respectively. After irradiation, no significant difference in the fraction of surviving cells was found between NN and FPN or SPN donors. Mean surviving fractions at 2 Gy (SF2) of NN, FPN, and SPN donors were 0.54 ± 0.16, 0.58 ± 0.16, and 0.53 ± 0.16, respectively.

### Chromosome Aberrations

#### G1 Assay

**Figure 2:**
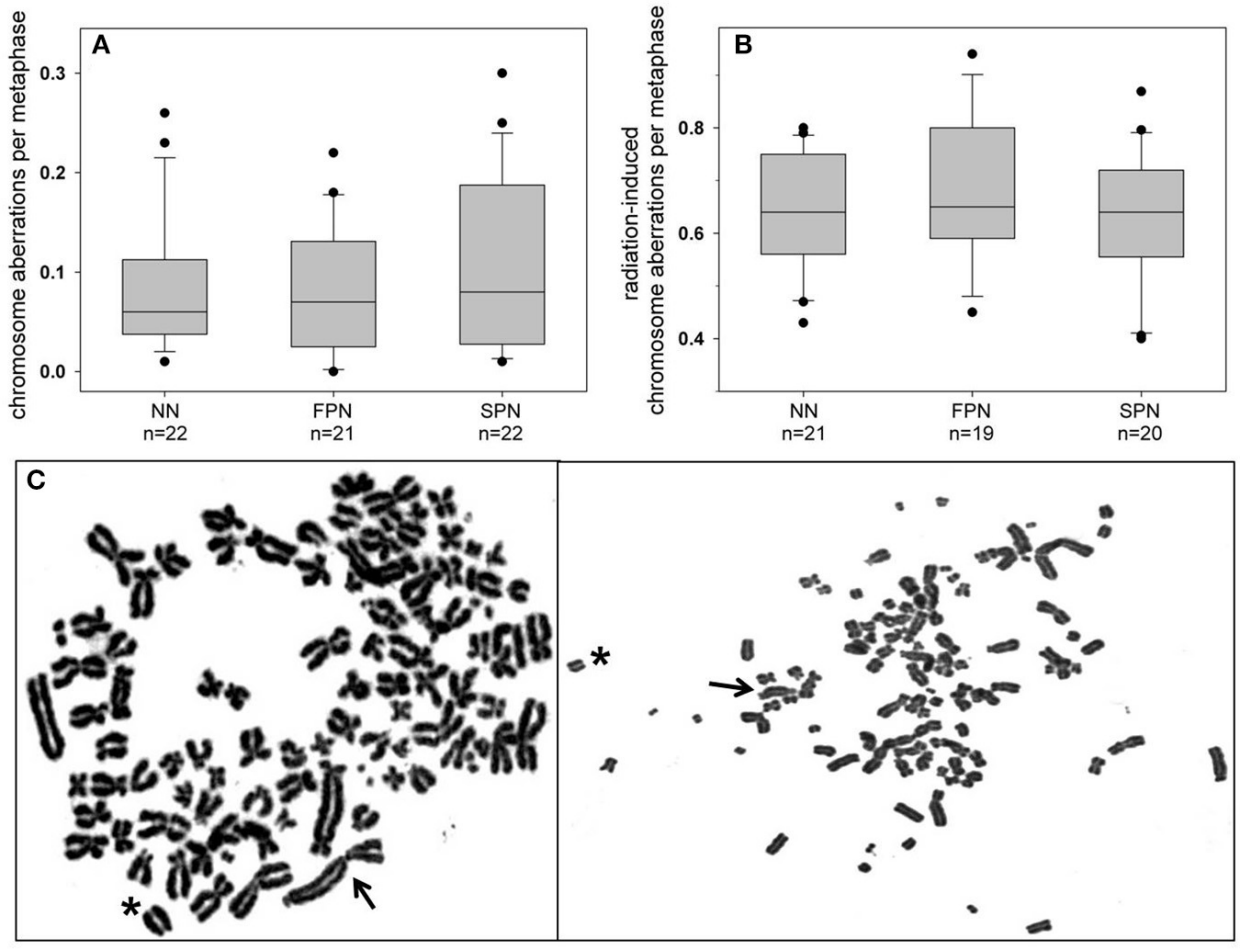
Box and whisker plots of **(A)** spontaneous and **(B)** radiation-induced chromosome aberrations in primary skin fibroblasts from donors with no neoplasm (NN), only a first primary neoplasm (FPN), or an FPN and a subsequent second primary neoplasm (SPN). Aberrations were scored in metaphases of the first cell cycle after exposure in G1 to 3 Gy X-rays. Boxes include 50% of the data. The inner line represents the median value, whiskers represent the minimum and maximum values, and circles mark outliers. **(C)** Two representative Giemsa-stained complete metaphases from an SPN donor showing an abnormally high rate of spontaneous numerical and structural aberrations. Differences in the morphology of chromosomes between metaphases result from the different degrees of condensation. Structural aberrations are exemplarily indicated (arrow: dicentric chromosome, asterisk: acentric fragment).

For the G1 assay, a total of 65 donors were analyzed for the rate of spontaneous chromosome aberrations, yielding a mean of 0.091 ± 0.073 aberrations per cell. Concerning the different study populations, the highest, although insignificantly elevated average rate of 0.103 ± 0.083 aberrations per cell was found in SPN donors compared to 0.083 ± 0.064 in FPN donors and 0.086 ± 0.068 in NN donors as shown in Figure 2A. For the different types of aberrations, the only divergence was a very mild increase of chromatid exchanges in SPN donors. Detailed information on the rate of chromosome aberrations scored in the G1 assay is provided in Table 3.

**Figure 3:**
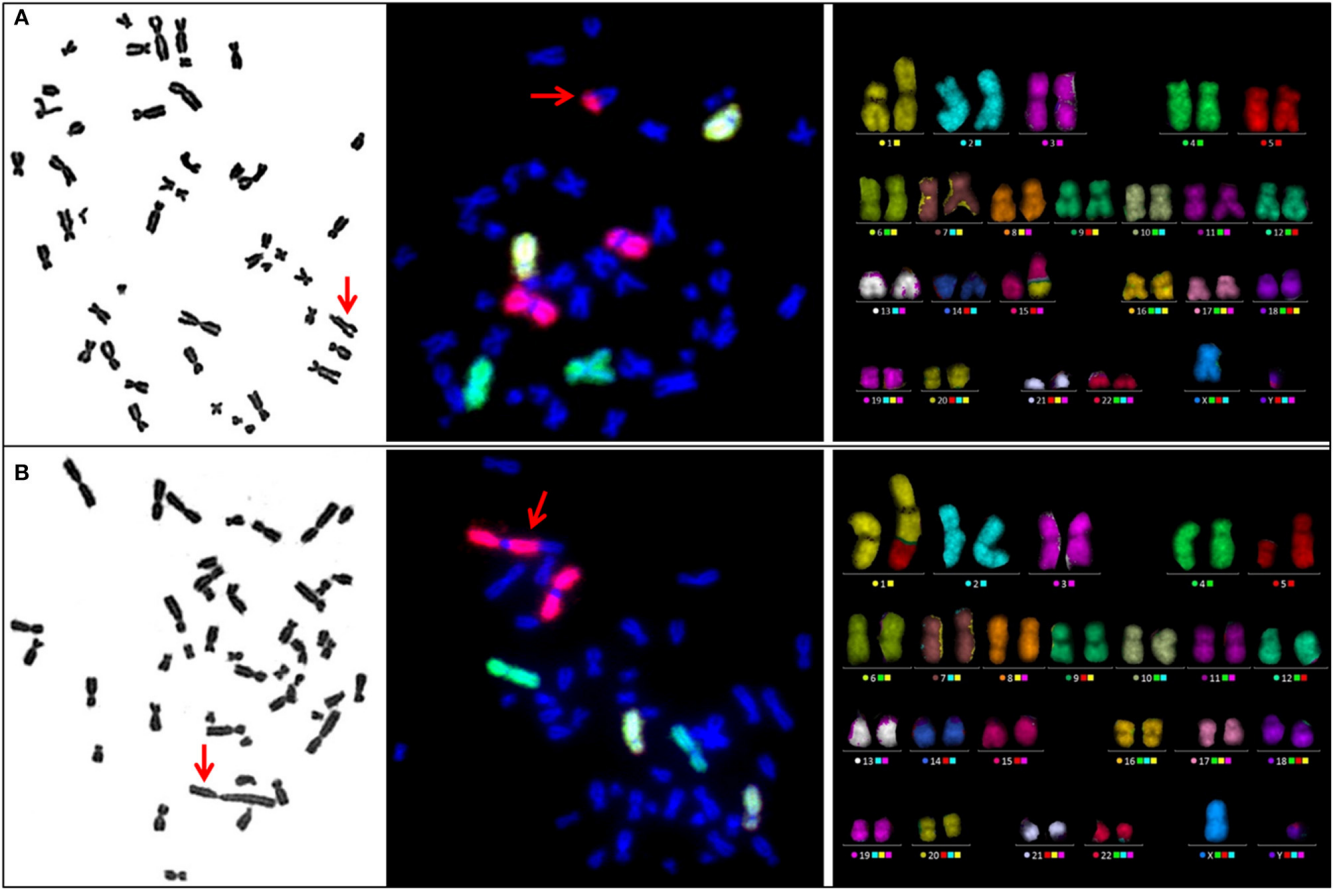
Representative metaphases of an SPN donor carrying the spontaneous clonal translocations **(A)** t (15′-1) (1′T) and **(B)** t (1′-5) (5′T) after Giemsa staining (left panel), three-color FISH (central panel, red: chr. 1, green: chr. 2, yellow: chr. 4) and mFISH (right panel). Translocations are indicated by red arrows in metaphase spreads after Giemsa staining and three-color FISH.

Remarkably, metaphase analysis of sham-irradiated cells revealed a striking chromosomal instability in two SPN donors. One SPN donor, who suffered from lymphoma as the FPN and SPN, showed exceptionally high numbers of numerical and unstable structural aberrations in 4 out of 100 analyzed metaphases. Representative pictures of such highly aberrant metaphases of this donor are shown in Figure 2C. Otherwise stable ploidy levels were observed for all other donors. The mean proportion of tetraploid cells in sham-irradiated samples of all donors was 1.68 ± 1.60% (range 0–8.8%) with no significant differences between the study populations (*p* = 0.703). Detailed information on tetraploidy is provided in Table 3. For a second SPN donor, Giemsa-analysis already indicated the presence of two clonal translocations as shown in Figure 3. Since this patient suffered from a pediatric rhabdomyosarcoma as the FPN which has been associated with the translocations (2;13)(q35;q14) or (1;13)(p36;q14) in tumor specimen (45), we screened all four donors of the respective quadruplet with two FPN cases for the involvement of chromosomes 1, 2, and 4 in cytogenetic alterations by three-color FISH. The NN donor and the two FPN donors showed no or a very low frequency of aberrations involving chromosome 1 in ≤ 3% of metaphases, whereas it was involved in aberrations in 84% of the metaphases of the SPN donor. Chromosomes 2 and 4 were not involved in aberrations in any donor analyzed. Subsequent mFISH analysis of the SPN donor revealed the following translocations: t (15′-1) (1′T) clonal in 53% of metaphases, t (1′-5) (5′T) clonal in 28% of metaphases, t (15′-21) (21′-15) clonal in 5% of metaphases, t (15‘-6) (6‘-15), t (11‘-8) (8‘T) clonal in 2% of metaphases as well as non-clonal translocations t (15′-1) (1′T) t (2′-12) (12′-2), and t (16′-17) (17′-16) in one metaphase each. In total 90% aberrant metaphases were detected by mFISH. The aberrations were found in different cell cultures and passages of cells from this donor which were performed for the extensive cytogenetic analyses. Representative pictures of aberrant metaphases and karyotypes of this SPN donor after Giemsa-staining, three-color FISH, and mFISH are shown in Figure 3 and Supplementary Figure S2.

After irradiation of fibroblasts with 3 Gy X-rays in G1 the mean yield of IR-induced chromosome aberrations in first post-exposure mitoses of a total of 61 donors was 0.650 ± 0.129 per cell. Shown in Figure 2B, the different sub-groups of donors had comparable average rates of IR-induced aberrations per cell of 0.642 ± 0.114 in NN donors, 0.683 ± 0.148 in FPN donors and 0.628 ± 0.124 in SPN donors. For a qualitative examination of the accuracy of DSB repair the average rates of RI interchromosomal exchanges scored as dicentric chromosomes were compared between the different donor groups. RI dicentrics occurred at similar frequencies per cell of 0.321 ± 0.062 in NN donors, 0.348 ± 0.065 in FPN donors, and 0.324 ± 0.065 in SPN donors.

#### G2 Assay

**Figure 4:**
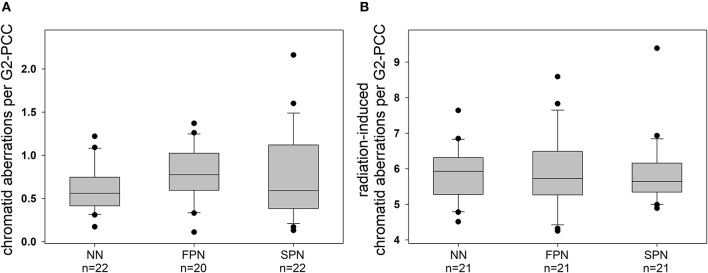
Box and whisker plots of **(A)** spontaneous and **(B)** radiation-induced chromatid aberrations in primary skin fibroblasts from donors with no neoplasm (NN), only a first primary neoplasm (FPN), or an FPN and a subsequent second primary neoplasm (SPN). Aberrations were scored in prematurely condensed chromosomes of G2 cells (G2-PCCs) 3 h after the exposure of exponentially growing cells to 1 Gy X-rays. Boxes include 50% of the data. The inner line represents the median value, whiskers represent the minimum and maximum values, and circles mark outliers.

The analysis of chromatid aberrations in G2-PCCs from a total number of 64 donors showed a mean yield of spontaneous aberrations of 0.706 ± 0.377 per cell, primarily attributed to the occurrence of chromatid breaks and gaps. The rates for the different sub-groups of donors were comparable with 0.615 ± 0.274 aberrations per cell in NN donors, 0.767 ± 0.321 in FPN donors, and 0.742 ± 0.495 in SPN donors (Figure 4A). Three hours after exposure to 1 Gy X-rays the average yield of IR-induced chromatid aberrations in 63 donors was 5.88 ± 0.921 per G2-PCC. The different study populations showed similar mean rates of IR-induced chromatid aberrations per G2-PCC amounting to 5.83 ± 0.791 in NN donors, 5.90 ± 1.08 in FPN donors, and 5.91 ± 0.982 in SPN donors (Figure 4B). Detailed information on the rate of chromatid aberrations scored in G2-PCCs is provided in Table 4.

### Correlations Between Assays and Patient Characteristics

Concerning a relationship between the results of the G1 and the G2 assay, only the level of spontaneous aberrations correlated weakly (*r* = 0.41, *p* < 0.001). No correlation was observed between the SF2 obtained in the clonogenic survival assay and the results of any cytogenetic evaluation. Statistical analysis did not reveal a significant impact of a previous RT, CT, gender, or tumor entity (solid vs. hematopoietic or lymphoid) as well as FPN or SPN on the average level of spontaneous or IR-induced chromosome aberrations in both assays. Only *in vitro* exposure to IR increased the probability for the formation of a chromosome aberration per cell significantly by 8.1- and 10.4-fold for the G1 and G2 assay, respectively. Details on statistical evaluations are provided in Supplementary Tables S1, S2.

## Discussion

With increasing success in tumor control due to the constant progress of diagnostics and therapeutic strategies in oncology, treatment-related adverse late-effects inevitably gain high clinical relevance. Iatrogenic high-grade toxicities and second primary malignancies are a major threat and cause of long-term morbidity for the continuously increasing number of cancer survivors, in particular for childhood cancer patients (1, 2, 6). The present study examined if a relation between the susceptibility to pediatric FPNs or therapy-related SPNs and impaired genome maintenance exists. Therefore, measurements of sporadic chromosomal instability and cellular or chromosomal IR sensitivity in normal somatic cells were performed in matched SPN, FPN, and NN donors. We observed no significant difference for clonogenic cell survival after IR or the average yield of spontaneous and IR-induced chromosome aberrations between the study populations. Striking spontaneous chromosomal abnormities were found in two donors with SPN only. The results obtained in this study population indicate that the etiology of sporadic childhood cancer or the risk for SPN might underlie limited DNA repair capacities and provide useful information for future studies including the need for other biomarkers.

Since intrinsic proneness to cancer has been closely related to alterations in the DNA damage response a variety of studies have been conducted to identify high-risk patients by using biomarkers of DNA damage and repair. Over the past two decades evidence accumulated that chromosomal IR sensitivity of lymphocytes assessed by the conventional analysis of metaphases after irradiation of cells in G2 may serve as an indicator for proneness to an FPN. Such correlations of an increased innate chromosomal IR sensitivity and cancer susceptibility have been discussed regarding the presence of unknown, low-penetrance predisposing genes, in particular for patients with early-onset malignancies (27, 46). Many studies used classification criteria based on the average yield or arbitrary thresholds for the dispersion of IR-induced chromosome aberrations in G2 lymphocytes obtained at diagnosis to stratify for normal and sensitive IR responders and showed higher fractions of IR sensitive patients with sporadic and familial histories of breast cancer (14–21, 24, 47), with brain tumors (22, 23), head and neck squamous cell carcinomas (26), colorectal cancer (24), or suffering from different tumor entities (25). Moreover, these results point toward higher fractions of IR sensitive individuals among young adults with early-onset cancers (14, 26). For pediatric FPNs, Baria et al. (27) and Curwen et al. (28) confirmed an increased chromosomal IR sensitivity of G2 lymphocytes from childhood cancer patients. Using bioassays to quantify DSBs visualized as foci of γH2AX, 53BP1, or pATM (phosphorylated ataxia telangiectasia mutated), Rube et al. (30) and Schuler et al. (31) reported a compromised repair of IR-induced DSBs in G0 lymphocytes of childhood cancer patients suffering from different tumor entities, most pronounced in patients developing life-threatening or even lethal normal-tissue toxicities. Their findings also emphasize the strong selection criterion and potential bias of our study by examining only long-term survivors of pediatric FPNs compared to the vast majority of studies conducted on lymphocytes drawn at the time of tumor diagnosis. Hitherto, a sole nested case-control study performed by Haddy et al. (29) investigated differences in the repair of IR-induced DSBs between childhood cancer patients with an FPN and patients with a subsequent SPN. Applying γH2AX fluorescence intensity measurements by flow cytometry as a surrogate marker for DSBs in patient-derived lymphoblastoid cell lines established on average 24 years after the diagnosis of the FPN, an association between higher rates of basal as well as IR-induced DSBs and the risk of SPN in childhood cancer survivors was demonstrated. However, no tumor-free controls were included in the study.

To the best of our knowledge, the nested case-control study presented here is the first comparing the intrinsic genome integrity as well as the cellular and cytogenetic response to IR in primary fibroblasts obtained from patients with an FPN who developed an SPN or not and matched tumor-free controls. The above-cited studies have been performed with primary peripheral blood lymphocytes or lymphoblastoid cell lines which might affect the comparability with the results obtained in fibroblasts in the present study. Even though systemic lymphocytes are by far more easily accessible by minimal invasive venepuncture, we have chosen to conduct our investigations on primary skin fibroblasts since the use of lymphocytes is fraught with some major drawbacks: (1) they are unsuitable for long-term conservation and propagation unless immortalization with the Epstein-Barr virus transformation is performed which has profound impacts on cell cycle regulation via the expression of viral oncogenes, (2) they are largely exposed to genotoxic anticancer drugs including irradiation of bone marrow during radiotherapy, (3) they are prone to hematopoietic mosaicisms, and (4) they are unsuitable after a bone marrow or stem cell transplantation. The latter applies in particular to the present study since seven former childhood cancer patients received a bone marrow or stem cell transplant during their course of cancer therapy. As known from various studies of donors with chromosomal instability syndromes like Fanconi anemia, Bloom or Nijmegen breakage syndrome, sporadic as well as clastogen-induced chromosome aberrations in lymphocytes can be very well recapitulated in different cell types of the same donor such as skin fibroblasts (48, 49) or buccal epithelial cells (50). In this field, the use of skin fibroblasts is even preferred for diagnostic purposes since the occurrence of hematopoietic mosaicisms can obscure germline mutations and generate false-negative results (48, 49). Furthermore, a study by Lobrich et al. (51) demonstrated that the intrinsic defect in DSB repair of a patient showing radiation hypersensitivity during radiotherapy could be detected qualitatively and quantitatively to a comparable extent in his lymphocytes and fibroblasts by γH2AX foci quantification after IR exposure. We are aware that the quantity and quality of IR-induced chromosome aberrations are dependent on various factors including nuclear geometry and architecture (52, 53) which differs between adherent and flat fibroblasts or spherical lymphocytes in suspension. However, this study did not aim to draw a direct quantitative comparison on the yield of IR-induced aberrations between previous studies using lymphocytes and our fibroblast data but to compare sporadic and IR-induced aberrations in the same cell type between the different donor groups. Using primary skin fibroblasts as a model of the normal somatic tissue we did not observe any difference in the average rate of spontaneous chromosome aberrations or clonogenic survival and aberrations after IR exposure between the study populations. Thus, our results show a comparable efficiency of genome maintenance between former pediatric patients with a high proneness to cancer *per se* or an SPN and tumor-free donors. Only a slight trend toward elevated spontaneous aberrations in SPN donors was observed with two SPN-cases displaying exceptional chromosomal instability. SPN donors showed a larger degree of variation in the clonogenic survival as well as for the level of spontaneous unstable aberrations in both assays when compared to FPN cases and tumor-free donors. Improving statistics by a larger cohort size or by increasing the number of evaluated cells through automated scoring of cytogenetic damage might corroborate these results in the future. Studies based on large epidemiological cohorts already showed an association between high levels of spontaneous unstable chromosome aberrations in peripheral blood lymphocytes of healthy individuals and cancer risk independent of previous exposures to carcinogens (32, 33, 54). Also, cytogenetic evaluations in somatic cells of pediatric cancer patients suggest that constitutional or treatment-related karyotype instability might promote the development of an SPN (54–56).

In this present study, the occurrence of aneuploid (near tetraploid) metaphases with a very high burden of structural aberrations in fibroblasts of an SPN donor who suffered from two independent lymphomas reflects a tumor-like karyotype and is a very clear indicator of exceptional chromosomal instability. Tetraploidization and a mild accumulation of aberrant metaphases can be a feature of *in vitro* aged primary fibroblasts approaching replicative senescence but are usually not found in recently established cultures at low passages as used here (57). Apart from the case described above, ploidy levels were normal with comparable fractions of tetraploid cells for all participants. Another SPN donor with a rhabdomyosarcoma as the FPN and a subsequent lymphoma as the SPN displayed high rates of clonal and non-clonal translocations in 90% of metaphases detected by high-resolution mFISH. Non-clonal translocations as well as their clonal expansion have been documented in skin fibroblasts from patients with hematopoietic malignancies after high-dose CT plus total-body RT before bone marrow transplantation (58). However, such aberrations can also occur as an artifact during the *in-vitro* cultivation of fibroblasts from normal donors (41). Here, RT was administered as partial-body irradiation and skin biopsies were taken outside the treatment field according to common RT plans of the enclosed tumor entities. Therefore, the observed translocations were generated most probably spontaneous *in vivo* or *in vitro*. Although the translocations detected in the SPN donor showed frequent participation of chromosomes 1 and 15, the involvement of at least 9 different chromosomes in multiple translocations shows a genome-wide chromosomal instability in his normal somatic cells. Sporadic structural and numerical chromosomal instability are hallmarks and drivers of carcinogenesis and have been attributed to dysfunctions in the mitotic checkpoint, DNA repair, and replication (59). However, for example, DNA damage generated by replicative stress differs substantially in signal transduction and repair pathways compared to DSBs caused by IR, the inductor of DNA-damage used in our study (60). Thus, approaches using a predictive functional assay with a singular end-point as a surrogate marker for cancer sensitivity bear a high risk of missing the actual affected gene-products and pathways which define innate cancer proneness (12).

In conclusion, our results do not support previous findings of overall elevated spontaneous or IR-induced chromosome aberrations in normal somatic cells of individuals with early and high cancer incidence. Striking cytogenetic abnormalities, suggesting an elevated tumor risk, were detected in two SPN donors, only. High-resolution cytogenetic analysis by FISH for all donors which allows much more sensitive detection of cytogenetic damage including transmissible aberrations that are missed by conventional solid-staining may sustain such findings in the future. Besides, testing the study population of this work for their proficiency to deal with replication stress-associated DNA damage induced by physical obstacles to the replication machinery is a future task to mimic and investigate the vulnerability to such pathophysiological processes related to cancer risk.

A drawback of this study is no intended matching of oncologic therapies between the corresponding FPN and SPN cases, in particular for RT, as it represents the highest risk factor for SPNs. Information on clinical management and medical histories of cancer patients has been provided only by the treating physicians voluntarily as well as on patient-based self-reports during a medical interview and is therefore very sparse and not standardized. Currently the cohort of the KiKme study is largely extended in a epidemiological nested case-control study design to obtain primary skin fibroblasts from 101 SPN and 340 FPN cases as well as from 150 tumor-free donors (Marron et al., in review) ^1^. Detailed questionnaires will provide information on lifestyle, socio-economical and anthropometric factors as well as on health, family history of diseases, and medical radiation history including phantom based dosimetry to obtain distinct organ doses. Detailed analysis of genetic predispositions and other molecular-biological factors is already underway. Next-generation sequencing approaches and functional assays on DNA repair in a high-throughput design will be performed to unravel risk factors and potential predictive biomarkers for childhood cancers and treatment-related second malignancies for the most benefit of future cancer patients.

## Data Availability Statement

All datasets generated for this study are included in the article/Supplementary Material.

## Ethics Statement

The studies involving human participants were reviewed and approved by Ethics Committee of the Medical Association of Rhineland-Palatinate [No. 837.440.03 (4102) and No. 837.262.12(8363-F)]. The patients/participants provided their written informed consent to participate in this study.

## Author Contributions

SZ, HS, MM, CS, DG, and ML: conception and design. SZ and HS: development of methodology. SZ, CH, DG, and LE: acquisition of biopsies and data. SZ, AP, CH, SR, and JM: analysis and interpretation of data (e.g., statistical analysis, biostatistics, and computational analysis). SZ: initial draft of the manuscript. SZ, AP, CH, TH, MM, JM, SR, PS-K, CS, and HS: writing, review, and/or revision of the manuscript. All authors contributed to the article and approved the submitted version.

## Conflict of Interest

The authors declare that the research was conducted in the absence of any commercial or financial relationships that could be construed as a potential conflict of interest.

## Footnotes

^1^Marron M, Brackmann L, Grandt C, Zahnreich S, Galetzka D, Schmitt I, et al. An innovative nested case-control study to identify genetic predispositions of childhood and second primary cancers related to ionizing radiation - the KiKme study. (in review).

## Abbreviations

SPN: second primary neoplasm
FPN: first primary neoplasm
NN: no neoplasm
CT: chemotherapy
RT: radiation therapy
DSB: DNA double-strand break
53BP1: tumor protein 53 binding protein 1
IR: ionizing radiation
G2-PCC: G2 premature chromosome condensation
FISH: fluorescence *in situ* hybridization
mFISH: 24-multicolor fluorescence *in situ* hybridization
t: translocation
ins: insertion
dic: dicentric chromosome
T: truncated
SF2: surviving fraction at 2 Gy
pATM: phosphorylated ataxia telangiectasia mutated.
