# Supplementary material for: Spontaneous and Radiation-Induced Chromosome Aberrations in Primary Fibroblasts of Patients With Pediatric First and Second Neoplasms
Source: Front Oncol. 2020 Aug 7;10:1338. doi: 10.3389/fonc.2020.01338 (PMC7427586; doi:10.3389/fonc.2020.01338)
Supplement: Supplementary file 1 [file Data_Sheet_1.PDF]

Supplementary Figure S1

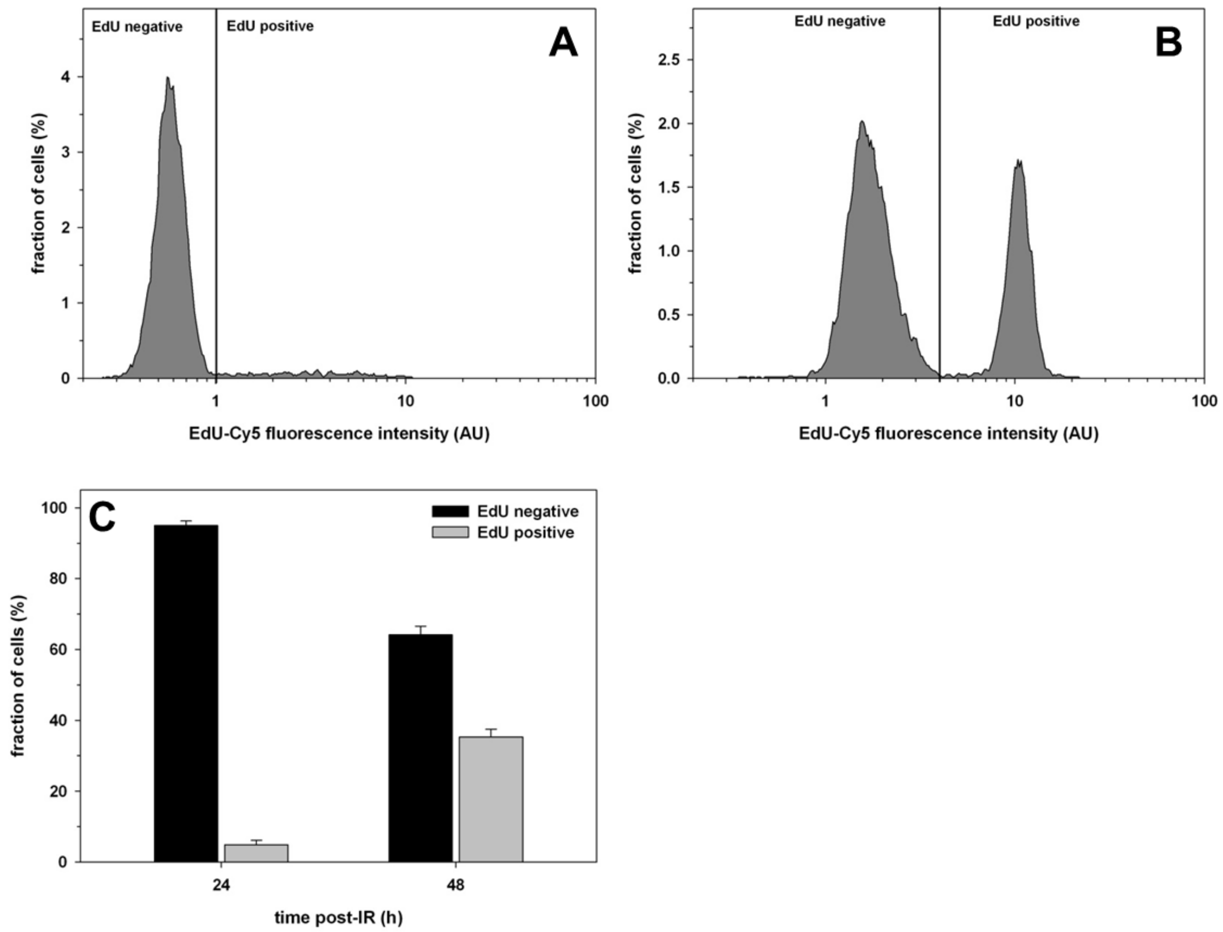

Supplementary Figure S1:
Cell cycle progression of G1-synchronized primary human fibroblasts after X-irradiation and delayed plating 24 h after exposure measured by cumulative EdU-labeling. Cells were grown for 14 days to reach confluency, irradiated with 3 Gy X-rays, and reseeded after 24 h for proliferation in the presence of 10 µM EdU (Lumiprobe) and 0.2 µg/ml colcemid (Roche) to label replicating cells and to prevent progression through the first mitosis post-exposure, respectively. After 24 h and 48 h cells were harvested, fixed in 70 % ethanol at -20° C and stained by EdU-click reaction kit according to the manufacturers' protocol (Roth). EdU-Cy5 fluorescence intensity was measured by flow cytometry (FACS Canto II^{TM}, Becton Dickison) in at least 10.000 cells per sample. Representative histograms of EdU-Cy5 fluorescence intensity 24 h and 48 h after delayed plating 24 h after exposure are depicted in (A) and (B), respectively. The mean fractions and standard deviations of EdU positive or EdU negative cells from biological triplicates out of one experiment are shown in (C). 24 hours after delayed plating, less than 5% of the cells showed proliferation activity and were positive for EdU incorporation. (AU, arbitrary units).

Supplementary Figure S2

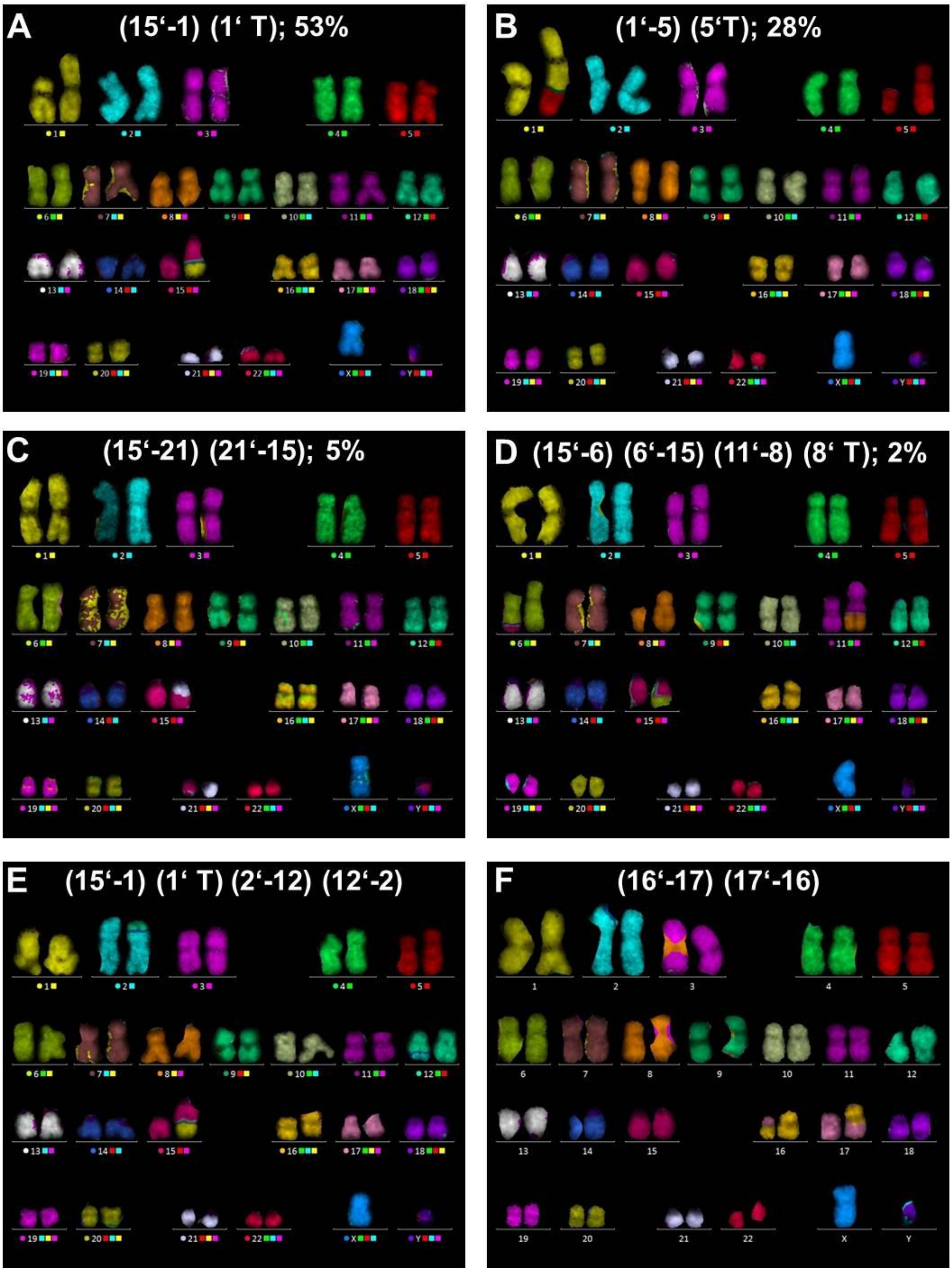

Supplementary Figure S2:

Aberrant karyotypes of sham-irradiated primary fibroblasts of an SPN donor after mFISH. Structural aberrations were classified according to the mPAINT system (42). Identical chromosome aberrations were termed clonal if they were present in at least two metaphases of one sample. Clonality occurred in (A-D) and the proportion of affected cells is provided in the respective karyotypes. (E) represents a karyotype with the clonal unbalanced translocation t (15’-1) (1’T) found in (A) plus an additional sporadic balanced translocation t (2’-12) (12’-2). (F) shows a sporadic balanced translocation (16’-17) (17’-16).

Supplementary Table S1: Results of the statistical analysis of chromosome and chromatid aberrations scored in the G1 assay using the R package glmmTMB.

	Estimate	Std. Error	z value	Pr (>|z|)	Exp (Estimate)
(Intercept)	-2.43379070	0.06124144	-39.7409098	0.0000000	0.08770374
Dose	2.08698258	0.04768845	43.7628482	0.0000000	8.06055636
Group FPN	-0.21775429	0.16959282	-1.2839829	0.1991479	0.80432305
Group SPN	-0.20509494	0.17266961	-1.1877883	0.2349168	0.81456998
CT	0.20849681	0.16361813	1.2742892	0.2025609	1.23182501
RT	0.06530024	0.07351898	0.8882093	0.3744282	1.06747947

Supplementary Table S2: Results of the statistical analysis of chromatid aberrations scored in the G2 assay using the R package glmmTMB.

	Estimate	Std. Error	z value	Pr (>|z|)	Exp (Estimate)
(Intercept)	-0.48249613	0.04230793	-11.4043886	3.975607e-30	0.6172408
Dose	2.34439500	0.02414362	97.1020525	0.000000e+00	10.4269625
Group FPN	0.13751882	0.13309004	1.0332766	3.014745e-01	1.1474233
Group SPN	0.12871198	0.13418400	0.9592200	3.374479e-01	1.1373625
CT	-0.05627467	0.12554589	-0.4482399	6.539801e-01	0.9452795
RT	-0.04041771	0.06205424	-0.6513287	5.148343e-01	0.9603882
